# Supplementary material for: GnRH Induces Citrullination of the Cytoskeleton in Murine Gonadotrope Cells
Source: Int J Mol Sci. 2024 Mar 10;25(6):3181. doi: 10.3390/ijms25063181 (PMC10970285; doi:10.3390/ijms25063181)
Supplement: Supplementary file 1 [file ijms-25-03181-s001.zip › Supplemental Figure S1/Supplementary Fig 1 (with figure legend).pptx]

## Slide 1
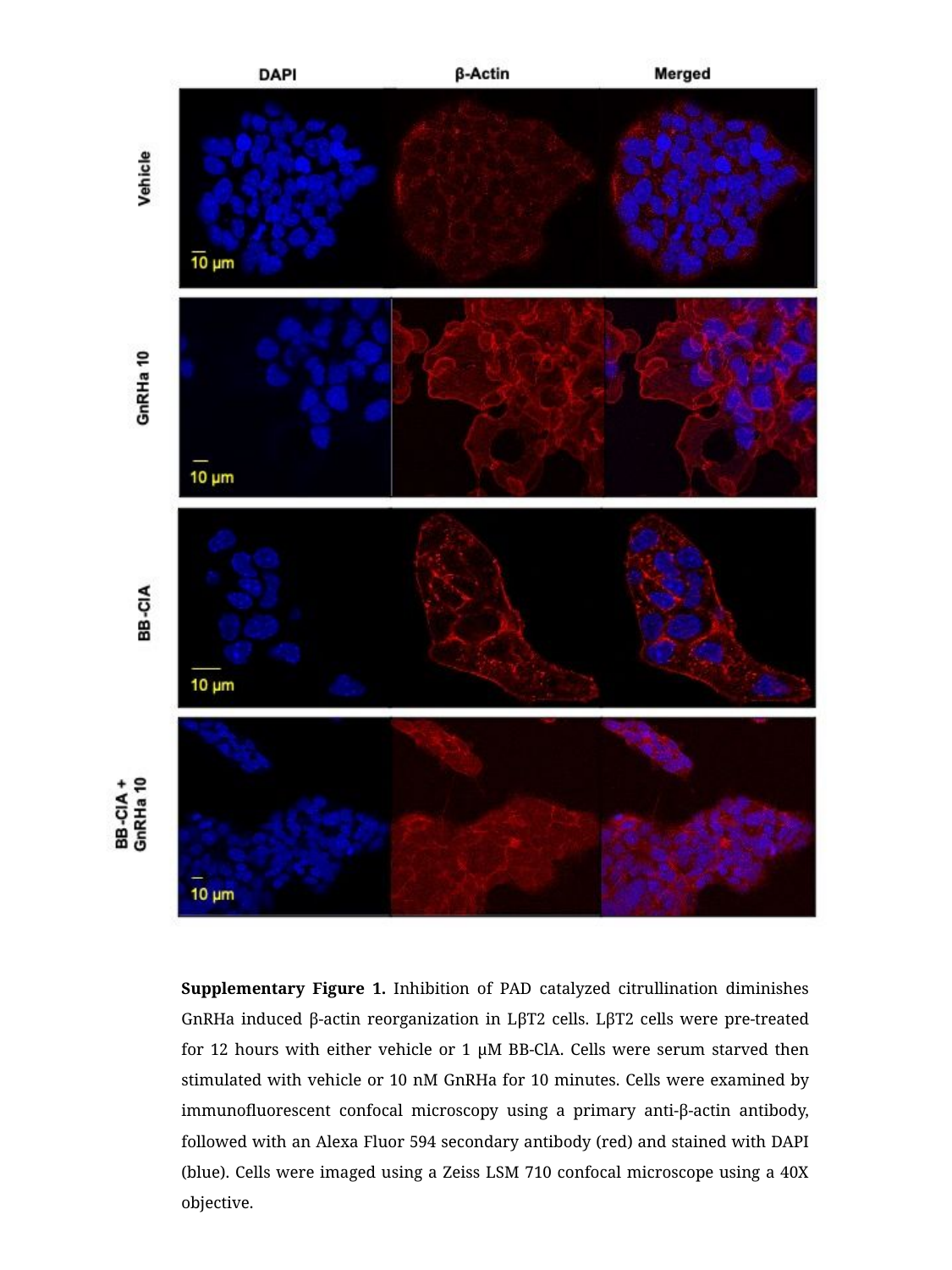

Supplementary Figure 1. Inhibition of PAD catalyzed citrullination diminishes GnRHa induced β-actin reorganization in LβT2 cells. LβT2 cells were pre-treated for 12 hours with either vehicle or 1 μM BB-ClA. Cells were serum starved then stimulated with vehicle or 10 nM GnRHa for 10 minutes. Cells were examined by immunofluorescent confocal microscopy using a primary anti-β-actin antibody, followed with an Alexa Fluor 594 secondary antibody (red) and stained with DAPI (blue). Cells were imaged using a Zeiss LSM 710 confocal microscope using a 40X objective.
