# Supplementary material for: GnRH Induces Citrullination of the Cytoskeleton in Murine Gonadotrope Cells
Source: Int J Mol Sci. 2024 Mar 10;25(6):3181. doi: 10.3390/ijms25063181 (PMC10970285; doi:10.3390/ijms25063181)
Supplement: Supplementary file 1 [file ijms-25-03181-s001.zip › Supplemental Figure S2/Supplementary Figure 2 Figure Legend.pptx]

## Slide 1
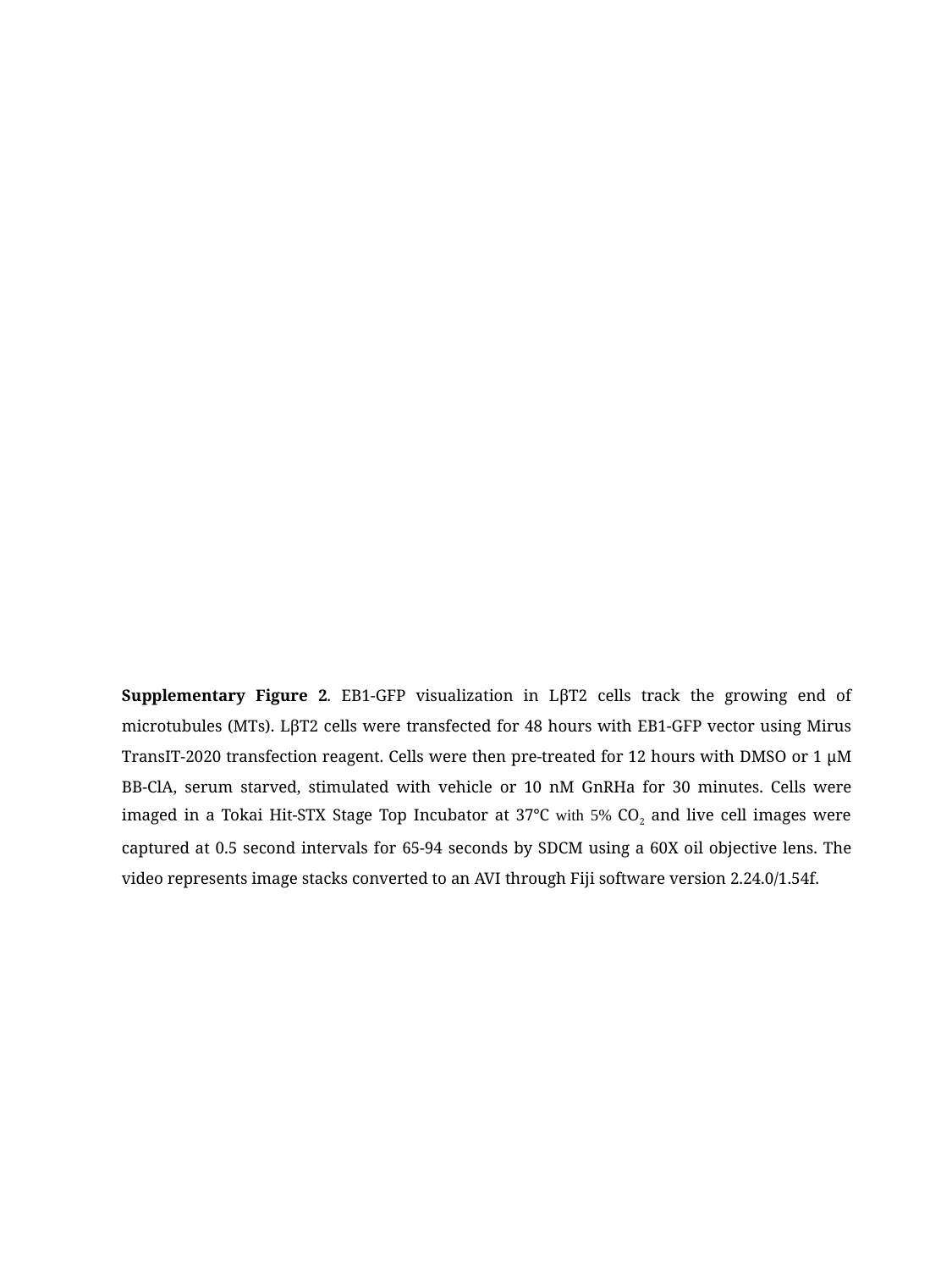

Supplementary Figure 2. EB1-GFP visualization in LβT2 cells track the growing end of microtubules (MTs). LβT2 cells were transfected for 48 hours with EB1-GFP vector using Mirus TransIT-2020 transfection reagent. Cells were then pre-treated for 12 hours with DMSO or 1 μM BB-ClA, serum starved, stimulated with vehicle or 10 nM GnRHa for 30 minutes. Cells were imaged in a Tokai Hit-STX Stage Top Incubator at 37℃ with 5% CO2 and live cell images were captured at 0.5 second intervals for 65-94 seconds by SDCM using a 60X oil objective lens. The video represents image stacks converted to an AVI through Fiji software version 2.24.0/1.54f.
