# Supplementary figures and images for: GnRH Induces Citrullination of the Cytoskeleton in Murine Gonadotrope Cells
Source: Int J Mol Sci. 2024 Mar 10;25(6):3181. doi: 10.3390/ijms25063181 (PMC10970285; doi:10.3390/ijms25063181)

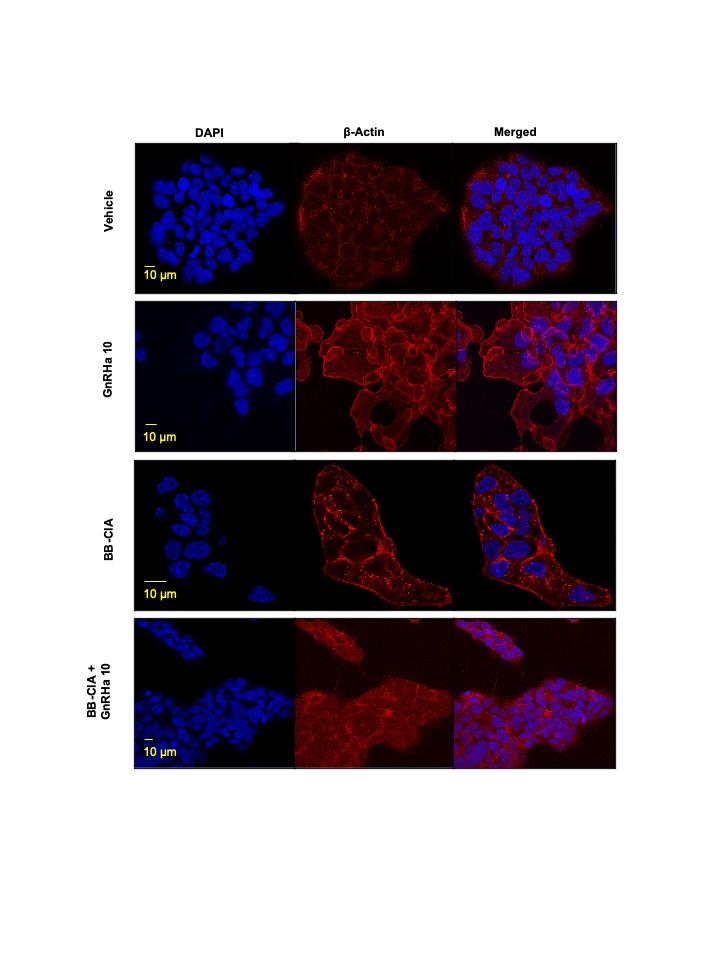

Supplement: Supplementary file 1 [file ijms-25-03181-s001.zip › Supplemental Figure S1/Supplementary Figure 1.jpg]
